# The Influence of Thermal Parameters on the Self-Nucleation Behavior of Polyphenylene Sulfide (PPS) during Secondary Thermoforming

**DOI:** 10.3390/ma17040890

**Published:** 2024-02-15

**Authors:** Yi Ren, Zhouyang Li, Xinguo Li, Jiayu Su, Yue Li, Yu Gao, Jianfeng Zhou, Chengchang Ji, Shu Zhu, Muhuo Yu

**Affiliations:** 1 State Key Laboratory for Modification of Chemical Fibers and Polymer Materials, Shanghai Collaborative Innovation Center of High-Performance Fibers and Composites (Province-Ministry Joint), Key Laboratory of High-Performance Fibers & Products, Ministry of Education, Center for Civil Aviation Composites, Donghua University, Shanghai 201620, China; renyidhu@163.com (Y.R.); 211100211@mail.dhu.edu.cn (Z.L.); lxg_0814@163.com (X.L.); 2210343@mail.dhu.edu.cn (J.S.); 11825055@zju.edu.cn (Y.G.); zjf@dhu.edu.cn (J.Z.); jicc@dhu.edu.cn (C.J.); 2 Key Laboratory of Shanghai City for Lightweight Composites, College of Materials Science and Engineering, Donghua University, Shanghai 201620, China; 3 School of Chemistry and Chemical Engineering, Frontiers Science Center for Transformative Molecules, Shanghai Jiao Tong University, Shanghai 201306, China; liyue0411@sjtu.edu.cn

**Keywords:** polyphenylene sulfide, self-nucleation, non-isothermal crystallization, secondary thermoforming

## Abstract

**Highlights:**

1. The influence of temperature parameters in two thermal cycles on the self-nucleation (SN) behavior was revealed.
2. The heating rate and processing melt temperature during the first thermal cycle exhibited a synergistic effect on the SN behavior.
3. Under appropriate conditions, both the first and second thermal cycles can generate SN behavior, but the mechanisms were different.

**Abstract:**

During the secondary thermoforming of carbon fiber-reinforced polyphenylene sulfide (CF/PPS) composites, a vital material for the aerospace field, varied thermal parameters profoundly influence the crystallization behavior of the PPS matrix. Notably, PPS exhibits a distinctive self-nucleation (SN) behavior during repeated thermal cycles. This behavior not only affects its crystallization but also impacts the processing and mechanical properties of PPS and CF/PPS composites. In this article, the effects of various parameters on the SN and non-isothermal crystallization behavior of PPS during two thermal cycles were systematically investigated by differential scanning calorimetry. It was found that the SN behavior was not affected by the cooling rate in the second thermal cycle. Furthermore, the lamellar annealing resulting from the heating process in both thermal cycles affected the temperature range for forming the special SN domain, because of the refined lamellar structure, and expelled various defects. Finally, this study indicated that to control the strong melt memory effect in the first thermal cycle, both the heating rate and processing melt temperature need to be controlled simultaneously. This work reveals that through collaborative control of these parameters, the crystalline morphology, crystallization temperature and crystallization rate in two thermal cycles are controlled. Furthermore, it presents a new perspective for controlling the crystallization behavior of the thermoplastic composite matrix during the secondary thermoforming process.

## 1. Introduction

Advanced thermoplastic composites have garnered extensive attention due to their noteworthy attributes including lightweight, high strength, excellent toughness, elevated damage tolerance, and recyclability [1,2]. Carbon fiber-reinforced polyphenylene sulfide (CF/PPS) composites, as a vital material for the aerospace field, exhibit a compelling cost-performance ratio, especially when compared to poly(ether-ether ketone) (CF/PEEK) composites, despite marginal disparities in mechanical performance. Utilizing the characteristics of PPS that can be melted and cooled repeatedly, innovative manufacturing approaches such as stamping forming [3,4,5], in situ consolidation [6,7,8], and induction welding [9,10,11] have been devised. These secondary thermoforming methods enable rapid fabrication of CF/PPS composites, consequently reducing production cycles and minimizing manufacturing costs.

The thermal history of CF/PPS composites in the secondary thermoforming is complicated, involving multiple melting and cooling processes. PPS, as the matrix of thermoplastic composites, is a semi-crystalline polymer. The crystallization behavior of PPS is significantly influenced by these processes. Although the crystallization and melting behaviors [12,13,14,15] of PPS have been extensively investigated, most of studies only focused on a single melt-cooling cycle for PPS [16,17,18,19,20], ignoring the potential influence of multiple thermal cycles on the crystallization behavior of PPS. Self-nucleation (SN) is a distinctive nucleation mechanism observed in semi-crystalline polymers under specific conditions of repeated thermal cycling. The generation of SN is due to the residual crystalline fragments or locally ordered region in the melt [21]. The SN behavior in crystallization process manifests as promoted polymer crystallization behavior without the addition of external heterogeneous surfaces, such as nucleating agents [22], resulting in an increase in crystallization temperature during non-isothermal crystallization processes [23,24]. Therefore, it is important to pay attention to the effect of multiple thermal cycles on PPS crystallization.

It is noteworthy that PPS exhibits a strong melt memory effect [16,25,26]. Even when melted at 40 °C above its melting point, the crystallization process still shows SN behavior. Furthermore, within the processing melt temperature range of 292–323 °C, the SN behavior of PPS remains unchanged with temperature variations [26]. A similar melt memory effect is observed in many copolymers [27,28,29,30,31,32] and some homopolymers [33,34,35,36,37,38]. They all demonstrate SN behaviors a few to several tens of degrees above their melting point. This pronounced melt memory effect could be attributed to either a unique molecular chain topology [29,39,40] or potent intermolecular interactions [41].

PPS has a distinct structure, with alternating phenyl rings and sulfur atoms, leading to numerous sulfur bonds in its molecular chain. Consequently, PPS displays considerable instability at elevated temperatures. Potential thermal degradation actions, like chain scission [42,43], branching [44] and crosslinking [45,46], can change the molecular chain structure and conformation in the PPS melt, leading to its pronounced melt memory effect and unique SN behavior. Therefore, it is crucial to understanding the effects of thermal parameters in multiple thermal cycle on the SN behavior of PPS, and discern how to influence the molecular chain conformation to control the SN behavior. Such insights can guide the selection of thermal protocols in specific manufacturing processes.

In this study, the crystallization and remelting behaviors of PPS across two thermal cycles were systematically investigated. Employing the DSC technique, the relationship between the various thermal parameters in two thermal cycles and the SN behaviors of PPS were explored. Notably, through a comprehensive analysis involving crystallization temperature, processing melt temperature and crystalline morphology, the significant impact of lamellae annealing on the SN behavior of PPS was illuminated. The synergistic relationship between heating rates and processing melt temperature in affecting the crystallization behavior of PPS was highlighted. Overall, this work will not only provide new insights into the SN behavior of PPS, but also provide a theoretical basis for the controllable processing of PPS and CF/PPS composites.

## 2. Materials and Methods

### 2.1. Materials

The material studied in this work is a commercial PPS film obtained from Zhejiang NHU Co., Ltd. (Shaoxing, China). It has a weight-average molecular weight (*M*_w_) of 48,200 g/mol, and a polydispersity index (PDI) of 4.03. The specified glass transition temperature is 87 °C and the melting point is 284.6 °C.

### 2.2. Characterizations

#### 2.2.1. Differential Scanning Calorimetry

The melting and crystallization behaviors were performed using a TA Instruments Q20 (TA instrument, New Castle, DE, USA) under a nitrogen atmosphere (50 mL/min). The instrument was calibrated before each set of scans with high-purity indium or tin. Samples (6.0 ± 0.5 mg) were sealed in aluminum pans.

The primary objective of this study is to investigate the influence of various thermal cycle parameters on the SN behavior and the crystallization behavior of PPS during two thermal cycles. These parameters include the heating rate (*H*_r1_) and processing melt temperature (*T*_s1_) during the first thermal cycle, as well as the heating rate (*H*_r2_), melting temperature (*T*_s_), and cooling rate (*C*_r1_) during the second thermal cycle. This section provides a brief overview of the thermal protocol corresponding to PPS undergoing melt-cooling. The detailed thermal protocols examining the impact of individual variables on crystallization behavior are presented in the Section 3.

**Figure 1:**
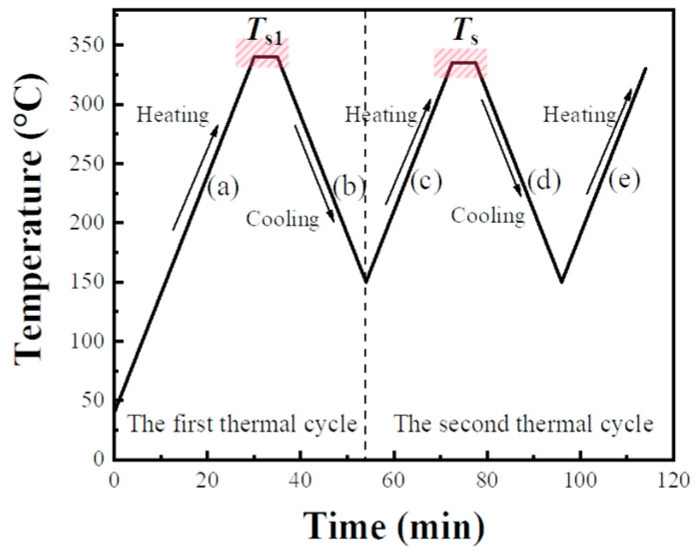
The thermal protocol used in DSC experiments. (a)–(e) respectively represent the heating and cooling stages during the thermal cycles.

The thermal protocol comprises two thermal cycles, and a reheating step for analyzing the PPS crystal structure, as shown in Figure 1.

For the first thermal cycle:

(a) Due to the broad melting range of PPS, the melting peak occurs between 250 °C and 290 °C. To ensure melting completely and eliminated any thermal history effects during the first melting process, the sample was heated to the initial processing melt temperature (*T*_s1_) at a specified heating rate (*H*_r1_), and held for 5 min. Incomplete melting due to a too-low *T*_s1_ would impact the consistency of the PPS melt during the first thermal cycle, ultimately affecting the investigation of PPS crystallization behavior.
(b) The sample was then cooled to 150 °C at a designated cooling rate, completing its initial crystallization.

For the second thermal cycle:

(c) The crystallized sample was subsequently reheated to the processing melt temperature (*T*_s_) at a specified heating rate (*H*_r2_), and held for 5 min. Different *T*_s_ led to distinct molecular chain conformations in PPS, subsequently resulting in varied SN behaviors and crystallization behaviors.
(d) The sample was cooled again to 150 °C at a specified cooling rate (*C*_r2_) for its second crystallization.
(e) Finally, the twice-crystallized sample was heated from 150 °C to 330 °C at a rate of 10 °C/min to analyze the crystalline structure forming in the second crystallization.

#### 2.2.2. Polarized Optical Microscopy

The crystalline morphology of PPS during crystallization was observed by Olympus BX-53 optical microscopes (Olympus Corporation, Shinjuku-ku, Japan) operating in reflection mode equipped with a Linkam THMS 600 hot stage (Linkam Scientific Instruments Ltd., Tadworth, UK). Images were taken with a Motic CCD camera, and for analysis of crystallization on cooling, NIH ImageJ software (Version 1.46 r) was used.

## 3. Results and Discussion

### 3.1. Effect of the Cooling Rate in the Second Thermal Cycle on the SN Behavior of PPS

**Figure 2:**
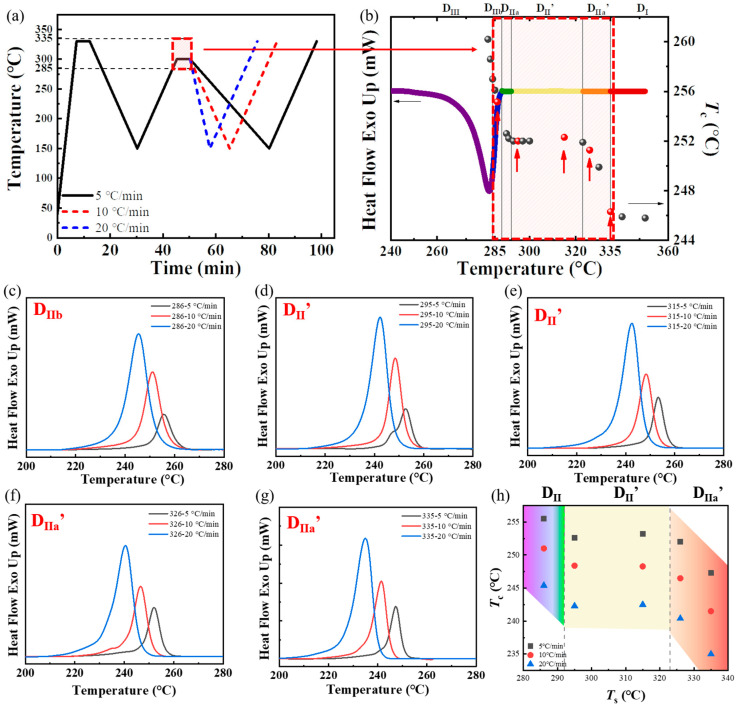
(**a**) The thermal protocol used by DSC, in which the cooling rates of the second thermal cycle is different. (**b**) The reference for the division of PPS Domain [26]. DSC cooling scans at different cooling rates when the secondary processing melt temperature (*T*_s_) is (**c**) 285 °C, (**d**) 295 °C, (**e**) 315 °C, (**f**) 325 °C, and (**g**) 335 °C. The corresponding SN domain was marked in the upper left corner, and the legend showed the corresponding cooling rate at the *T*_s_. (**h**) Peak crystallization temperature (*T*_c_) as a function of the *T*_s_ with different cooling rates. The colors of the shadows correspond to the temperature ranges associated with the different domains of PPS SN behavior.

During the secondary thermoforming of PPS, the molecular chain conformation in the melt exhibits variations based on the processing melt temperature. Specifically, with an elevation in processing melt temperature, the conformation transitions sequentially through stages: from residual crystalline fragments to locally ordered regions of molecular chains, followed by relaxation, interdiffusion, re-entanglement, and eventually reverts to a fully disordered random coil conformation [26]. It is worth noting that, due to differences in entropy, these various conformations of molecular chains showcase different crystallization temperature upon cooling. Additionally, the cooling rate is a significant factor affecting the non-isothermal crystallization behavior of semi-crystalline polymers, profoundly influencing the nucleation and subsequent crystal growth behaviors [47,48,49,50,51,52]. Therefore, it is imperative to focus on the influence of varying cooling rates on crystallization temperature of PPS when PPS exhibits SN behaviors. The thermal protocol applied to PPS was illustrated in Figure 2a.

The SN behavior of PPS is greatly influenced by the secondary processing melt temperature. In our previous studies [26], we observed distinct SN behaviors of PPS at different secondary processing melt temperatures, which we divided into six domains, as shown in Figure 2b. Each domain represented by a different colored line on the PPS melting enthalpy curve. Domain II_b_ represents PPS melting within a specific temperature range, where residual crystalline fragments remain in the melt. These fragments act as self-seeds and promote the nucleation process during cooling. Domain II_a_ indicates PPS melting within a temperature range where crystalline fragments are absent and are replaced by locally ordered molecular chains. This configuration can promote the nucleation process in PPS. Notably, PPS has a unique region called Domain II’. Within this domain, the crystallization temperature remains constant despite the increase in processing melt temperature, and maintaining a high nucleation density. This domain forms because the molecular chains in PPS cannot interdiffuse within this temperature range due to the complex molecular chain topology of the PPS amorphous region and the strong interaction between molecular chains. PPS is in Domain II_a_’ when the melting temperature is sufficient for interdiffusion of PPS molecular chains through thermal motion. In this domain, as the melting temperature continues to increase, the self-nucleation behavior of PPS gradually disappears. Based on prior research, five typical secondary processing melt temperatures (*T*_s_) were selected for applying two thermal cycles to PPS. Each of these temperatures falls within specific ranges that can induce various SN behaviors in PPS. These temperatures are marked with red dots in Figure 2. A temperature of 285 °C corresponds to Domain II_b_. For Domain II’, two temperatures were selected: 295 °C corresponds to the lower temperature of this domain, while 315 °C represents the higher temperature. A temperature of 326 °C corresponds to Domain II_a_’, and 335 °C corresponds to the temperature region where the SN effect almost disappears. After melting at these varied temperatures, three different cooling rates were used to finish non-isothermal crystallization during the PPS cooling process.

As shown in Figure 2c–g, it is evident that with a constant *T*_s_, the crystallization peak shifted to a lower temperature with an increase in cooling rate. Figure 2h illustrates a declining trend in the peak crystallization temperature (*T*_c_) of PPS within Domain II_b_ as the *T*_s_ increased. When the *T*_s_ was in Domain II’, the *T*_c_ of PPS remained unaltered with variations in *T*_s_, even if the cooling rate was different. As the *T*_s_ continued to increase, the *T*_c_ of PPS decreased again. This could be attributed to the diminishing melting memory effect, caused by the PPS molecular chains gradually reverting to a random coil conformation. Notably, although a higher cooling rate resulted in an overall decrease in crystallization temperature, the exhibited trend—a decline, followed by stabilization, and a subsequent decline—with respect to *T*_s_ variations remains consistent across different crystallization rates.

The consistency in the trend of *T*_c_ and *T*_s_ shown between different cooling rate groups in Figure 2h indicated that the molecular chain conformations associated with different SN behaviors of PPS were highly stable in the melt. This signifies that, while the cooling rate can alter the crystallization temperature, the nucleation mechanism during the crystallization process is not affected by the cooling rate. Furthermore, the constant *T*_c_ maintained in Domain II’ across different cooling rates suggested that the molecular chain conformation of PPS is consistent within this temperature range. Even if melt temperature differed by 20 °C, the molecular chains in the melt all maintain this particular metastable state. Consequently, theses conformation exhibited the same efficacy in fostering nucleation and facilitating the growth of spherulites, resulting in the same crystallization temperature within Domain II’.

### 3.2. Effect of the Heating Rate in the Second Thermal Cycle on the SN Behavior of PPS

**Figure 3:**
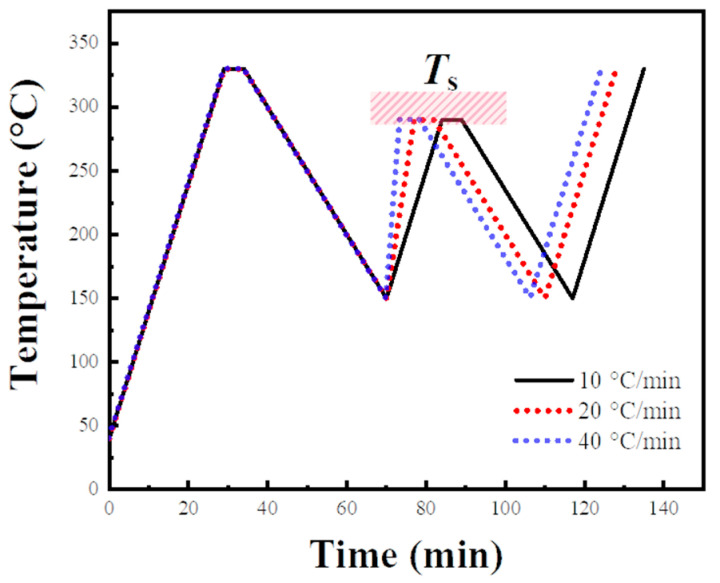
Thermal protocol used in experiment of investigating the relationship between the crystallization behavior and heating rate in the second thermal cycle. The range of the secondary processing melt temperature (*T*_s_) was from 283 °C to 350 °C, and the stay period at *T*_s_ was 5 min.

As the crystals are heated, they undergo a process known as annealing. This process could further promote lamellae growth, leading to a more perfect internal crystalline structure [53,54,55]. For CF/PPS composites during secondary thermoforming, there exists a significant difference in heating rates due to diverse processing method. Such variation in heating rate might result in different annealing processes for the PPS spherulites. This can potentially lead to varying degrees of perfection in the PPS crystalline structure, ultimately influencing the SN behavior of PPS. As depicted in Figure 3, heating rates of 10 °C/min, 20 °C/min, and 40 °C/min were chosen to examine the impact of different heating rates in the second thermal cycle on the SN behavior of PPS.

**Figure 4:**
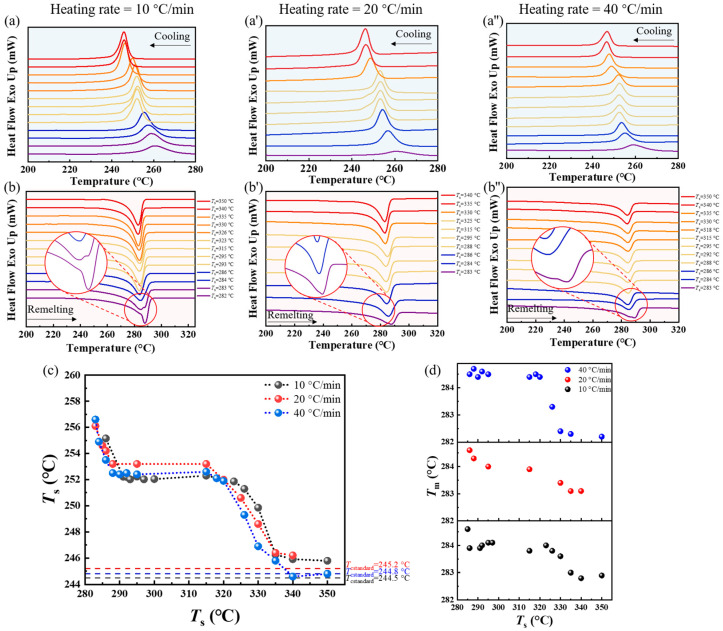
DSC cooling scans from the indicated *T*_s_ during the second thermal cycle, when the secondary heating rate was set to (**a**) 10 °C/min, (**a′**) 20 °C/min, and (**a″**) 40 °C/min. Different line colors represented different domains where PPS was located. Subsequent DSC heating scans which correspond to the third heating segments. The secondary heating rate was set to (**b**) 10 °C/min, (**b′**) 20 °C/min, and (**b″**) 40 °C/min. (**c**) Peak crystallization temperature (*T*_c_) as a function of *T*_s_ for different heating rates during the second thermal cycle. The dashed line represents the standard crystallization temperature of PPS. (**d**) Peak remelting temperature (*T*_m_) after crystallization in the second thermal cycle as a function of *T*_s_.

From Figure 4a–a″, it can be observed that the trend in crystallization temperature (*T*_c_) changes with *T*_s_ remained largely consistent across different secondary heating rates. Initially, as *T*_s_ increased, the crystallization peak shifted to a lower temperature. This trend sustained until a specific temperature was reached, after which the *T*_c_ plateaued, maintaining this equilibrium until approximately 320 °C. Beyond this *T*_s_, the *T*_c_ of PPS declined once more, which then remained constant at a higher temperature. The Using a heating rate of 20 °C/min for illustration: At *T*_s_ of 283 °C, the *T*_c_ descended from 256.1 °C to 253.2 °C with increasing *T*_s_. Subsequently, once *T*_s_ surpassed 288 °C, the *T*_c_ remained steady at 253.2 °C, persisting until 325 °C. When *T*_s_ exceeded 325 °C, the crystallization peak once again trended towards a lower temperature, ultimately stabilizing beyond 340 °C. From Figure 4b–b″, it can be noted that when *T*_s_ was 283 °C, samples with different heating rates exhibited double peaks in the remelting curves after second crystallization. This is the typical characteristic representation of Domain III. It means that at this temperature, the PPS spherulites were only partially melted, and the unmelted part was annealed.

To clearly analyze the influence of the heating rate on the SN behavior, the relationship between the *T*_c_ and the *T*_s_ under different heating rates was compiled into Figure 4c. It can be observed that the heating rate did not significantly affect the relationship between *T*_s_ and corresponding *T*_c_. Furthermore, regardless of the heating rate, the special region (Domain II’) consistently emerged between 292 °C and 318 °C. Within this region, the *T*_c_ did not change with an increase in the *T*_s_. This phenomenon further validated the stable existence of this region. However, it should be noted that an increase in the heating rate caused Domain II’ to shift overall to lower temperature. When the heating rate was 10 °C/min, the *T*_s_ range of domain II’ is defined between 292 °C and 323 °C, which was based on the relationship between the *T*_s_ and *T*_c_. However, when the heating rate was 20 °C/min and 40 °C/min, respectively, this domain began at 288 °C, and the *T*_c_ no longer changed with the increase in *T*_s_. Only when the *T*_s_ exceed 320 °C/min, the *T*_c_ decreased again with the increasing *T*_s_.

The change in the temperature region of Domain II’ suggests that the SN behavior of PPS is indeed influenced by different heating rates in the second thermal cycle. Analyzing the *T*_c_ between groups with different heating rates reveals that despite the heating rates varied, the *T*_c_ within Domain II’ remained almost consistent. This indicates that the molecular chain conformation in the melt prior to crystallization is essentially the same, and therefore the promotion effect on post-melting crystallization is nearly identical. When PPS spherulites undergo a secondary heating process, annealing occurs and the lamellae thicken. Additionally, during the annealing process, various defects contained within the PPS crystalline regions are gradually expelled such as branched chains, impurities, and various dislocations [56]. This makes the topological structure of the amorphous molecular chains between the crystalline regions even more complex. When the heating rate is slow, the lamellar have sufficient time to anneal and become thicker. Therefore, a higher temperature is required to destroy the more perfect residual crystalline fragments and locally ordered regions. It also requires a higher temperature to break the metastable state where the molecular chains are relaxed but do not interdiffuse. Conversely, faster rates suppress the annealing process and the lamellar thickening behavior, such as 20 °C/min. This allows PPS molecular chains to reach a metastable state at a lower processing melt temperature, ultimately destroying the metastable state at a lower temperature. Furthermore, it can be inferred from Figure 4d that the SN behavior of PPS leads to a discernible rise in the peak remelting temperature of the crystals. As this SN behavior gradually diminished with increasing *T*_s_, there was a corresponding gradual reduction in the peak remelting temperature, which eventually kept stable.

### 3.3. Effect of the Heating Rate in the First Thermal Cycle on the SN Behavior of PPS

The premise of SN behavior in semi-crystalline polymers is that the polymer needs to be sufficiently crystallized before the secondary melting. In the actual secondary thermoforming process of CF/PPS composites, the raw material may either be amorphous or in a fully crystallized state, potentially leading to inconsistencies in the initial crystallization behavior of PPS. In this study, the PPS film is amorphous, and cold crystallization will occur during its first heating process. Then, the lamellae thicken and become perfect due to annealing during the heating process. Compared with the melt crystallization, the crystals of PPS with imperfect structure resulting from cold crystallization are more significantly affected by annealing. Hence, the amorphous PPS raw material might be strongly influenced by the heating rate in the first thermal cycle. Figure 4 have found that there were subtle differences in the memory effect of PPS melt under different heating rates in the second thermal cycle. Therefore, it is of interest to see if applying different heating rates in the first thermal cycle to the amorphous PPS film will affect its initial crystallization behavior, and finally change the SN behavior during the second crystallization of PPS.

**Figure 5:**
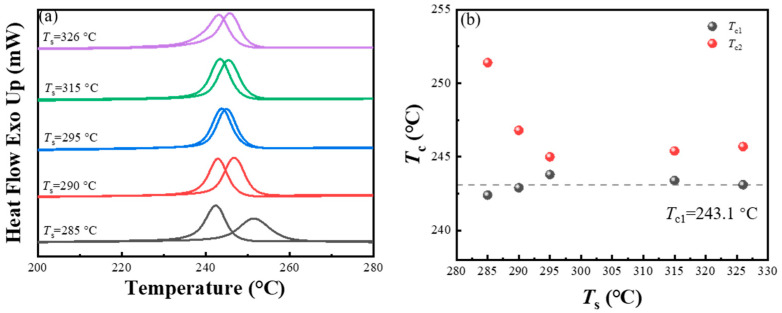
(**a**) DSC cooling scans during the first and second thermal cycles. The corresponding secondary processing melt temperature (*T*_s_) is marked on the left side of the curve. (**b**) Peak crystallization temperature as a function of the *T*_s_, which corresponds to the first (*T*_c1_) and second (*T*_c2_) cooling segments, respectively. The heating rate in the first thermal cycle was set to 10 °C/min, and the initial processing melt temperature was set to 330 °C.

The PPS was subjected to a heating rate of 10 °C/min and maintained at 330 °C for 5 min in the first thermal cycle. After cooling process, the temperature was increased again to observe the changes in the relationship between crystallization temperature (*T*_c2_) and the processing melt temperature (*T*_s_) in the second thermal cycle. As shown in Figure 5a, it was found that when *T*_s_ below 295 °C, the *T*_c2_ of PPS consistently decreased with the rise of *T*_s_. This is consistent with the SN behavior exhibited by PPS in previous experiments. As the *T*_s_ increased, diminishing residual crystalline fragments in the melt weakened the promotion effect of the self-seeds on PPS crystallization. However, for *T*_s_ exceeding 295 °C, the *T*_c2_ dropped to around the initial crystallization temperature (*T*_c1_). This phenomenon is quite unique. According to the previous experimental results of the SN behavior of PPS, *T*_s_ should be within the temperature range that could form PPS’s special SN domain (Domain II’). In this range, while the *T*_c2_ of PPS does not change with the increasing *T*_s_, the *T*_c2_ of PPS at this range should be higher than the *T*_c1_. This is because although PPS might lack residual crystalline fragments or ordered structural regions, its intricate amorphous chain topology restricts the interdiffuse of molecular chains. This particular conformation, with its inherent low entropy, promote the nucleation behavior of PPS. The phenomenon depicted in Figure 5b, as suggested by other literature reports, indicates that the memory effect of the polymer melt has disappeared and the SN behavior no longer occurs. At this temperature, the PPS melt was homogeneous, and nucleation was trigger on the foreign pre-existing surface provided by high-temperature-resistant heterogeneities [56].

**Figure 6:**
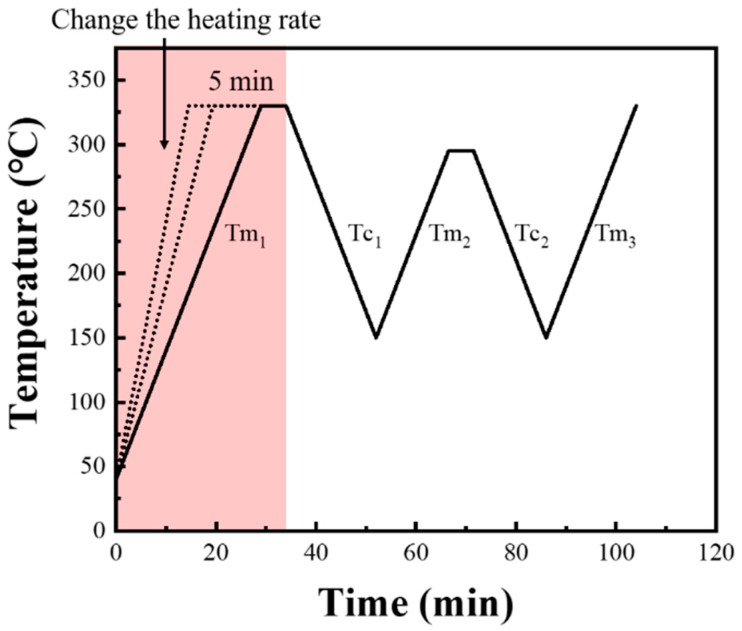
Thermal treatment process used in experiment of investigating the relationship between the crystallization behavior and the first heating rate.

Interestingly, as depicted in Figure 5b, with a heating rate of 10 °C/min in the first thermal cycle, the *T*_c1_ of PPS was 243.1 °C. However, when the first heating rate was escalated to 40 °C/min, under the same processing melt temperature and cooling rate, the *T*_c1_ of PPS was 240.5 °C. The slower first heating rate resulted in a higher *T*_c1_ for PPS during the initial crystallization. This suggests that the first heating rate had an impact on the *T*_c1_. Could this result explain the phenomenon that *T*_c2_ approached *T*_c1_ is not due to the disappearance of the SN effect, but rather because the *T*_c1_ increased due to the slow first heating rate? To further analyze the impact of the initial heating rate on both primary crystallization behavior and behavior after secondary melting, experiments were conducted while maintaining the same first processing melt temperature as before, which is 330 °C. The heating rates were set to 5 °C/min, 10 °C/min, 15 °C/min, 20 °C/min, and 40 °C/min. The thermal protocols were shown in Figure 6. With the processing melt temperature in the second thermal cycle selected as 295 °C, the thermal flow changes in the sample during thermal cycling at different heating rates were recorded.

**Figure 7:**
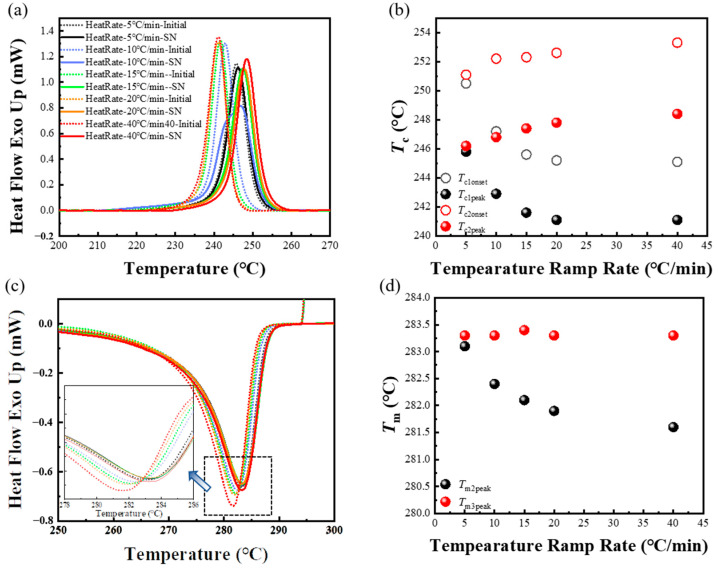
(**a**) DSC cooling scans and (**b**) onset crystallization temperature (*T*_conset_) and peak crystallization temperature (*T*_cpeak_) as a function of the temperature ramp rate, which corresponds to the first and secondary cooling segments, respectively. The selected secondary processing melt temperature was 295 °C. (**c**) The original DSC heating scans and (**d**) onset remelting temperature (*T*_monset_) and peak remelting temperature (*T*_mpeak_) as a function of heating rate in the first thermal cycle, which corresponds to the remelting process of spherulites forming in the initial and second crystallization, respectively.

As seen in Figure 7a, the varying first heating rates profoundly influenced both initial crystallization behavior and the second crystallization behavior. Notably, when the initial heating rate was 5 °C/min, the cooling curve of the initial cooling process almost coincides with that of the secondary cooling process. However, as the initial heating rate accelerated, the curves corresponding to the first and second crystallization process gradually separated. The first crystallization peak shifted towards a lower temperature, while the second crystallization peak shifted towards a higher temperature. Eventually, when the heating rate exceeded 20 °C/min, the relative positions of the two curves attained stability. The relationship between the crystallization temperature of the two stages and the initial heating rate was summarized in Figure 7b. It can be seen that when the first heating rate was 5 °C/min, the crystallization temperatures of the two stages were almost the same. As the heating rate increased, the *T*_c1_ gradually decreased, from 245.8 °C to 241.1 °C. In contrast, the *T*_c2_ gradually increased, from 246.2 °C to 248.0 °C. This resulted in an increasing temperature difference between the crystallization temperature of the two stages. When the heating rate was 40 °C/min, compared with the sample at a heating rate of 20 °C/min, the variances of crystallization temperature in both crystallization process were minimal, maintaining a steady temperature gap. It is crucial to highlight that although the initial processing melt temperature is the same, a slower heating rate resulted in a higher crystallization temperature (~4 °C). This suggests that there were some ordered structures in the melt that promoted the crystallization process of PPS, and these structures were formed at the slower heating rate.

Beyond the evident shifts in crystallization temperature, the impact of the initial heating rate on PPS crystallization behavior can also observe from the change in the remelting behavior of spherulites forming in the prior and subsequent cycles, as shown in Figure 7c,d. With the increases in the first heating rate, the peak remelting temperature of the spherulites forming in initial crystallization gradually decreased, dropping from 283.1 °C to 282.2 °C. In contrast, the peak remelting temperature of the spherulites forming in second crystallization remained relatively unchanged. It is noteworthy that the trend of the peak remelting temperature of the initial crystallization with the initial heating rate was almost the same as the trend of the peak melting temperature exhibited by the different SN behavior of PPS. The peak melting temperature could reflect the structural information of lamellae. According to Gibbs–Thomson equation [57], a higher peak remelting temperature corresponds to a more perfect lamellar structure. As can be seen from Figure 4d, if PPS displays SN behaviors during the cooling process after secondary melting, the peak remelting temperature of the formed crystals would be higher than that of regular spherulites. This is because PPS melt, which can generate SN behavior, can start to crystallize at a higher temperature during non-isothermal crystallization. The molecular chains move and arrange thoroughly at high temperature, leading the lamellae to become more orderly and perfected. As a result, it requires a higher temperature to melt these perfect lamellae. Thus, the static peak remelting temperature of spherulites forming in the subsequent crystallization, as depicted in Figure 7d, indicated that the crystalline structure of the second crystallization was not affected by the heating rate in the first thermal cycle.

It is worth noting that since there was SN behavior during the second crystallization process when the first heating rate was 40 °C/min. It could be speculated that SN behavior also occurred during the second crystallization when the first heating rate was 5 °C/min, due to the same *T*_m3peak_. Moreover, a consistent initial and second crystallization temperature at this heating rate suggested the same crystallization behaviors. Thus, based on the nearly identical crystallization temperature and peak remelting temperature in the two thermal cycles at a slower initial heating rate, it could be deduced that PPS exhibited SN behavior during the first crystallization.

**Figure 8:**
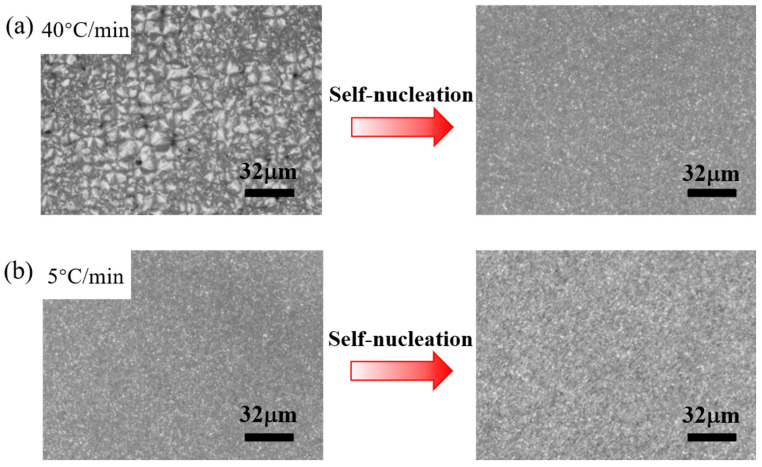
The change in PPS crystalline morphology in the first thermal cycle and the second thermal cycle. (**a**) The initial heating rate is 40 °C/min. (**b**) The initial heating rate is 5 °C/min.

To verify that under different heating rates in the first thermal cycle, PPS exhibits various crystallization behavior during the first crystallization due to the influence of cold crystallization and annealing. The same thermal protocol as DSC was applied to PPS films using a hot stage. The crystalline morphology was observed in real time by microscopy. Figure 8a shows that when the heating rate was 40 °C/min, the crystalline morphology of the initial crystallization manifested as standard spherulites. When the sample underwent a secondary melting and cooling, the SN behavior occurred, and the crystalline morphology was transformed into tiny crystals. However, at a heating rate of 5 °C/min, the initial crystalline morphology already presented as very tiny crystals, as shown in Figure 8b. This is the typical crystalline morphology following the SN behavior of PPS, where the volume of the spherulite dropped to a level that is difficult to discern with the naked eye. When this sample underwent the secondary melting and crystallization, the crystalline morphology is similar to that of the initial crystallization.

This result directly confirms that at a slower heating rate, PPS exhibits SN behavior during the initial crystallization process, leading to a significant increase in the crystallization temperature of PPS and a drastic reduction in crystalline size. This is due to the cold crystallization of the amorphous PPS film when it was heated in the first thermal cycle, and the crystals formed in the cold crystallization process underwent annealing during the slow heating process. This annealing process thickened the lamellae, perfected their structure, and expelled the various defects, thus forming a complex molecular chain topology between the lamellae. When the initial processing melt temperature was 330 °C, while the temperature can melt the annealed crystals, the complex molecular chain topology between the crystalline regions prevented the locally ordered relaxation molecular chains, originated from crystalline region, from reverting to the random coil conformation. Consequently, due to the advantage of entropy, the nucleation process of PPS is promoted by ordered molecular chains, manifesting as an increase in crystallization temperature and a sharp decrease in spherulitic size. However, when the heating rate surpasses 20 °C/min, the annealing process is suppressed, and the crystalline structure is not fully perfected. Thus, 330 °C could disrupt the metastable structure where molecular chains can relax but cannot interdiffuse. Ultimately, due to the absence of melt memory effect, PPS forms standard spherulites during cooling process. Additionally, the imaging results indicated that in situations where crystalline morphology cannot be obtained, the correlation between crystallization temperature and post-crystallization peak remelting temperatures could qualitatively predict crystalline morphology of PPS under given thermal conditions.

### 3.4. Effect of the Processing Melt Temperature in the First Thermal Cycle on the SN Behavior of PPS

**Figure 9:**
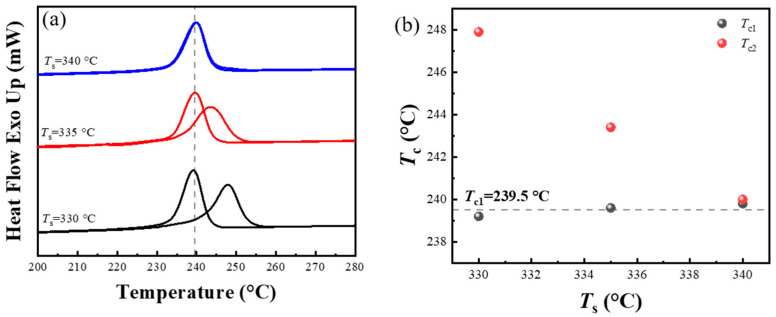
(**a**) DSC cooling scans during the first and second thermal cycles. The corresponding secondary processing melt temperature (*T*_s_) is marked on the left side of the curve. (**b**) Peak crystallization temperature as a function of the *T*_s_, which corresponds to the cooling segments in the first (*T*_c1_) and second thermal cycle (*T*_c2_), respectively. The first heating rate was set to 10 °C/min, and the initial melting rate was set to 340 °C.

The slow heating rate induces the annealing and thickening of lamellae in the PPS, which consequently elevates the processing melt temperature required for the polymer chains to return to the random coil conformation. This phenomenon’s implications for PPS processing are often understated in practical applications, which might lead to heightened crystallization temperature and increased viscosity during cooling process. To avoid the SN behavior occurring in the first thermal cycle, a higher processing melt temperature was used to eliminate the strong melt memory effect in PPS. By heating PPS at rate of 10 °C/min, annealing and thickening of the lamellae occurred after cold crystallization of PPS. The first processing melt temperature was then set to 340 °C. It can be seen from Figure 9a that when the processing melt temperature of second thermal cycle (*T*_s_) was set to 330 °C, the crystallization peak is separated from the crystallization peak of the first thermal cycle. As *T*_s_ increased, the crystallization peak began to shift toward low temperature. Finally, the crystallization peaks of the first and second thermal cycles were coincided when *T*_s_ increased to 340 °C. Figure 9b shows that after PPS melting at 340 °C in the first thermal cycle, the *T*_c1_ dropped to 239.5 °C, a reduction of 3.6 °C compared to the *T*_c1_ when the initial processing melt temperature was 330 °C. Importantly, the cooling rate for both sets were the same. Furthermore, it is noteworthy that a reemergence of the discrepancy between *T*_c1_ and *T*_c2_ was observed during the second thermal cycle of PPS. The *T*_c2_ was approximately 8 °C higher than the *T*_c1_, which is a typical manifestation of the SN behavior. As the processing melt temperature in the second thermal cycle (*T*_s_) increased, the *T*_c2_ gradually decreased due to the diminishing melt memory effect.

The experiment with an initial processing melt temperature of 340 °C also demonstrated the synergistic effects of the first heating rate and the initial processing melt temperature. A slower heating rate could induce a strong melt memory effect, which could be counteracted by elevating the initial processing melt temperature. To clearly reveal the impact of the single factor of initial processing melt temperature on the SN behavior in the second crystallization process, the heating should be set above 20 °C/min. This helps to reduce the influence of annealing and thickening during the initial heating process on the melt memory effect. Although the literature has widely reported the impact of the initial processing melt temperature on the crystallization temperature [58,59,60], it is important to focus on that whether it affects the crystallization behavior in the second thermal cycle.

**Figure 10:**
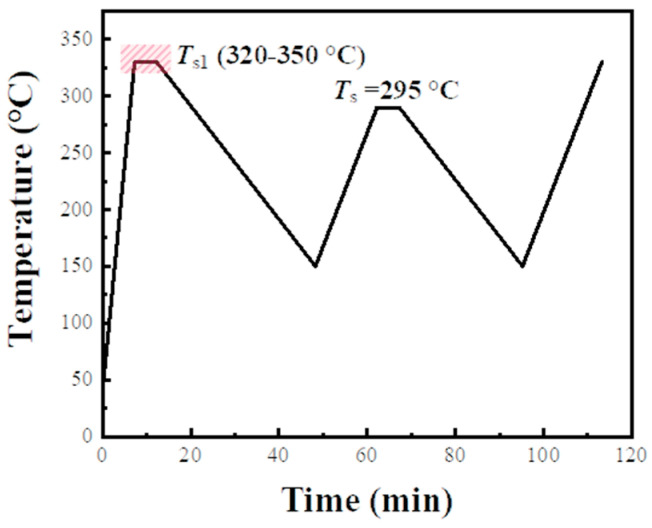
Thermal treatment process used in experiment of investigating the relationship between the crystallization behavior and initial processing melt temperature in the first thermal cycle.

**Figure 11:**
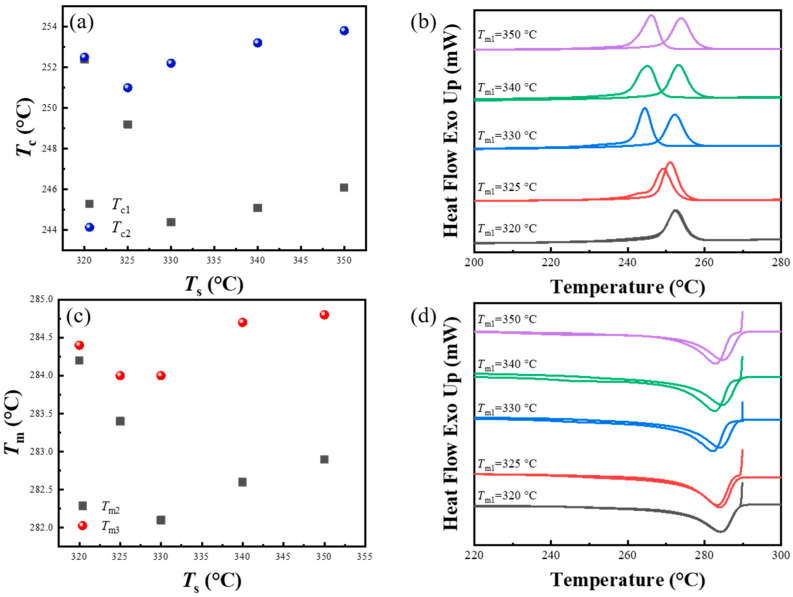
(**a**) Peak crystallization temperature as a function of the selected initial processing melt temperature (*T*_s1_), which corresponds to the cooling segments in the first (*T*_c1_) and second thermal cycle (*T*_c2_), respectively. (**b**) The original DSC cooling scans corresponding to the cooling process in two thermal cycle. The corresponding *T*_s1_ is marked on the left side of the curve. The secondary processing melt temperature was set to 295 °C. (**c**) Peak remelting temperature as a function of the *T*_s1_, which corresponds to the remelting process of spherulites forming in the initial (*T*_m2_) and second crystallization (*T*_m3_), respectively. (**d**) The original DSC heating scans.

The thermal protocol that was used to study the relationship between crystallization behaviors and initial processing melt temperature is shown in Figure 10. It can be seen from Figure 11a that when the initial processing melt temperature of PPS was below 330 °C, the initial crystallization temperature decreased from 252.4 °C to 244.4 °C with an elevation in the initial processing melt temperature. This is due to the melt memory effect caused by the PPS spherulites formed during cold crystallization, and the insufficient initial processing melt temperature cannot dimmish the melt memory effect. Although the annealing and thickening after cold crystallization is suppressed by fast heating rates, a suitably high processing melt temperature is essential to diminish the melt memory effect. In the thermal protocol corresponding to Figure 10, this proper initial processing melt temperature (*T*_s1_) was 330 °C, which corresponding to the lowest initial crystallization temperature (*T*_c1_). Beyond this processing melt temperature, the *T*_c1_ of PPS slightly increased with the increase in *T*_s1_, potentially due to minor crosslinking of molecular chains occurring within PPS.

Interestingly, when PPS underwent the second thermal cycle to induce the SN behavior, the *T*_c2_ was consistently around 252.4 °C. Figure 11b reveals that although PPS underwent melting at different temperature in the first thermal cycle, the heat flow curves during the secondary cooling process were almost identical, with no significant changes in peak shapes or positions. This consistency suggests minimal influence from the first processing melt temperature on second crystallization behavior with the SN effect. Furthermore, Figure 11c demonstrates that as the initial processing melt temperature gradually increased, the peak remelting temperature of the spherulites forming in the initial crystallization exhibited the same variation trend as the crystallization temperature. Notably, when the initial processing melt temperature was 320 °C, the crystallization and peak remelting temperature from both the prior and subsequent cycles were nearly the same, as shown in Figure 11d. Correspondingly, the heat flow curves during the cooling process and the heating process for spherulites remelting almost overlapped, indicating the occurrence of the SN behavior in both thermal cycles.

## 4. Conclusions

This study systematically investigated the crystallization and remelting behaviors of PPS during two thermal cycles. The effects of various conditions in the thermal cycles on the SN and crystallization behaviors of PPS were established.

(1) While the cooling rate in the second thermal cycle changed the crystallization temperature of PPS, it did not influence the SN behavior of PPS, which was manifested as the same temperature range of the special SN domain, as well as the consistent change trend between the processing melt temperature and crystallization temperature in the second thermal cycle across different crystallization rates.
(2) The heating rate of the second thermal cycle has a marginal influence on the perfection of the crystal. During the heating process of spherulites to melting, a slower heating rate led to significant annealing and thickening of lamellae, such as a rate less than 10 °C/min. As a result, the overall temperature range required for the formation of the specific Domain II’ in PPS was lower by approximately 4 °C.
(3) When PPS undergoes the first thermal cycle, there is a synergistic effect between the heating rate and the processing melt temperature. At a slower heating rate or a lower processing melt temperature, such as a rate less than 10 °C/min or a first-cycle processing melt temperature below 330 °C, PPS displays SN behavior. The SN behavior observed in the first thermal cycle originates from crystals formed during the cold crystallization of PPS. These crystals are annealed, and the lamella thickening at a lower heating rate. Additionally, the defect excluded from the lamella might heighten the existing entanglement of the molecular chains. This results in PPS requiring a higher temperature to revert to the random coil conformation. In addition, even with a faster heating rate like 40 °C/min, if the processing melt temperature is too low and insufficient to disrupt the locally ordered regions of the PPS molecular chains, it will also lead to a strong melt memory effect. Therefore, controlling the SN behavior of PPS requires simultaneous control of these two factors.

In summary, this study revealed the temperature parameters necessary for the initiation or inhibition of SN behavior in PPS during two thermal cycles. Furthermore, it elucidated the impact of each temperature parameter on PPS SN behavior, providing novel insights to control the crystallization temperature and morphology of PPS during secondary thermoforming processes.

## Data Availability

Data are contained within this article.
